# Ion Selectivity of Water Molecules in Subnanoporous Liquid‐Crystalline Water‐Treatment Membranes: A Structural Study of Hydrogen Bonding

**DOI:** 10.1002/anie.202008148

**Published:** 2020-10-19

**Authors:** Ryusuke Watanabe, Takeshi Sakamoto, Kosuke Yamazoe, Jun Miyawaki, Takashi Kato, Yoshihisa Harada

**Affiliations:** ^1^ Department of Advanced Materials Science Graduate School of Frontier Sciences The University of Tokyo 5-1-5, Kashiwanoha, Kashiwa Chiba 277-8561 Japan; ^2^ Department of Chemistry and Biotechnology School of Engineering The University of Tokyo 7-3-1, Hongo, Bunkyo-ku Tokyo 113-8656 Japan; ^3^ Institute for Solid State Physics (ISSP) The University of Tokyo 5-1-5, Kashiwanoha, Kashiwa Chiba 277-8581 Japan

**Keywords:** hydrogen bonding, liquid crystals, membranes, water nanochannels, X-ray emission spectroscopy

## Abstract

We demonstrate hydrogen‐bonded structures of water in self‐organized subnanoporous water treatment membranes obtained using synchrotron‐based high‐resolution soft X‐ray emission spectroscopy. The ion selectivity of these water treatment membranes is usually understood by the size compatibility of nanochannels in the membrane with the Stokes radius of hydrated ions, or by electrostatic interaction between charges inside the nanochannels and such ions. However, based on a comparison between the hydrogen‐bonded structures of water molecules in the nanochannels of the water treatment membrane and those surrounding the ions, we propose a definite contribution of structural consistency among the associated hydrogen‐bonded water molecules to the ion selectivity. Our observation delivers a novel concept to the design of water treatment membranes where water molecules in the nanochannel can be regarded as a part of the material that controls the ion selectivity.

## Graphical abstract

Hydrogen‐bonded structures of water in a self‐organized subnanoporous water treatment membrane obtained using soft X‐ray emission spectroscopy indicate that structural consistency among associated hydrogen‐bonded water molecules contributes to ion selectivity. MgSO_4_ with high permeability (73 %) has hydrogen‐bonded structures of hydrating water closer than those of NaCl with low permeability (30 %) to those in the water‐treatment membrane.

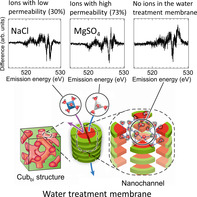

Membrane technologies for producing fresh water from seawater or brackish water have attracted attention because separation can be done with relatively low energy and a simple process.^[1]^ The separation technologies of reverse osmosis (RO) and nano‐filtered membranes have been developed.[^2^, ^3^, ^4^] Recently, self‐organized nano‐ or subnanoporous liquid‐crystalline (LC) membrane materials[^5^, ^6^, ^7^, ^8^] with uniform pore diameter size have been reported to show unique properties for water treatment[^5^, ^7^, ^8^] while for normal RO and nano‐filtered membranes the pore sizes are distributed. In the self‐organized LC membranes, a consistent size of water channels having a subnanometer to nanometer scale is formed by self‐assembly of columnar (Col) or bicontinuous cubic (Cub_bi_) LC monomers, which are preserved by in situ polymerization.[^5^, ^6^, ^7^, ^8^] The nanopores have a diameter ranging from 0.6 to 2.2 nm depending on the design of the self‐assembled and monomer chemical structures.[^7^, ^8^] To achieve more efficient separation properties and have deeper insight into the behavior of water molecules in such materials, it is important to study the hydrogen‐bonded structures of the molecules in the nanochannels.

For the Col and Cub_bi_ LC membranes obtained by polymerization of an ionic monomer, it is very interesting that a divalent sulfate ion (SO_4_^2−^) with larger ionic radius permeates the membranes more effectively than a monovalent ion (Cl^−^).^[7]^ For example, the permeability of MgSO_4_ is 73±5 %, which is more than double the 30±6 % value for NaCl. The mechanism of selective ion permeation is generally explained by the relationship between the sizes of ions and pores, which is typically the case for ion removal in a composite RO membrane. However, this behavior cannot be explained by the sieving effect and implies the presence of another driving force for ion selectivity. He and co‐workers discussed that apart from the size of ion hydration nuclei relative to the pore size, the energy required for dehydration plays an essential role in the ion selectivity of bioinspired graphene nanopores.^[9]^ Kawakami and co‐workers reported that the electrostatic interaction of water molecules with water treatment membranes affected the permeability of water.^[10]^ These discussions are consistent with MD simulations applied for different sizes of nanochannels,[^11^, ^12^, ^13^, ^14^] where the determinant factors for the ion selectivity are the pore size, end groups in the nanopores, membrane surface charge, and properties of ion hydration.

In the present study, we investigated hydrogen‐bonded structures of water in the nanostructured water treatment LC membranes formed by in situ polymerization of compound **1** (Figure 1 a) with synchrotron‐based high‐resolution X‐ray emission spectroscopy (XES) (Figure 1 b). We demonstrate the hydrogen‐bonded configuration of hydrating water of the ionic solute to understand the microscopic mechanism of the selective ion permeation of monovalent chloride and divalent sulfate ions in LC membranes with subnanopores.

**Figure 1:**
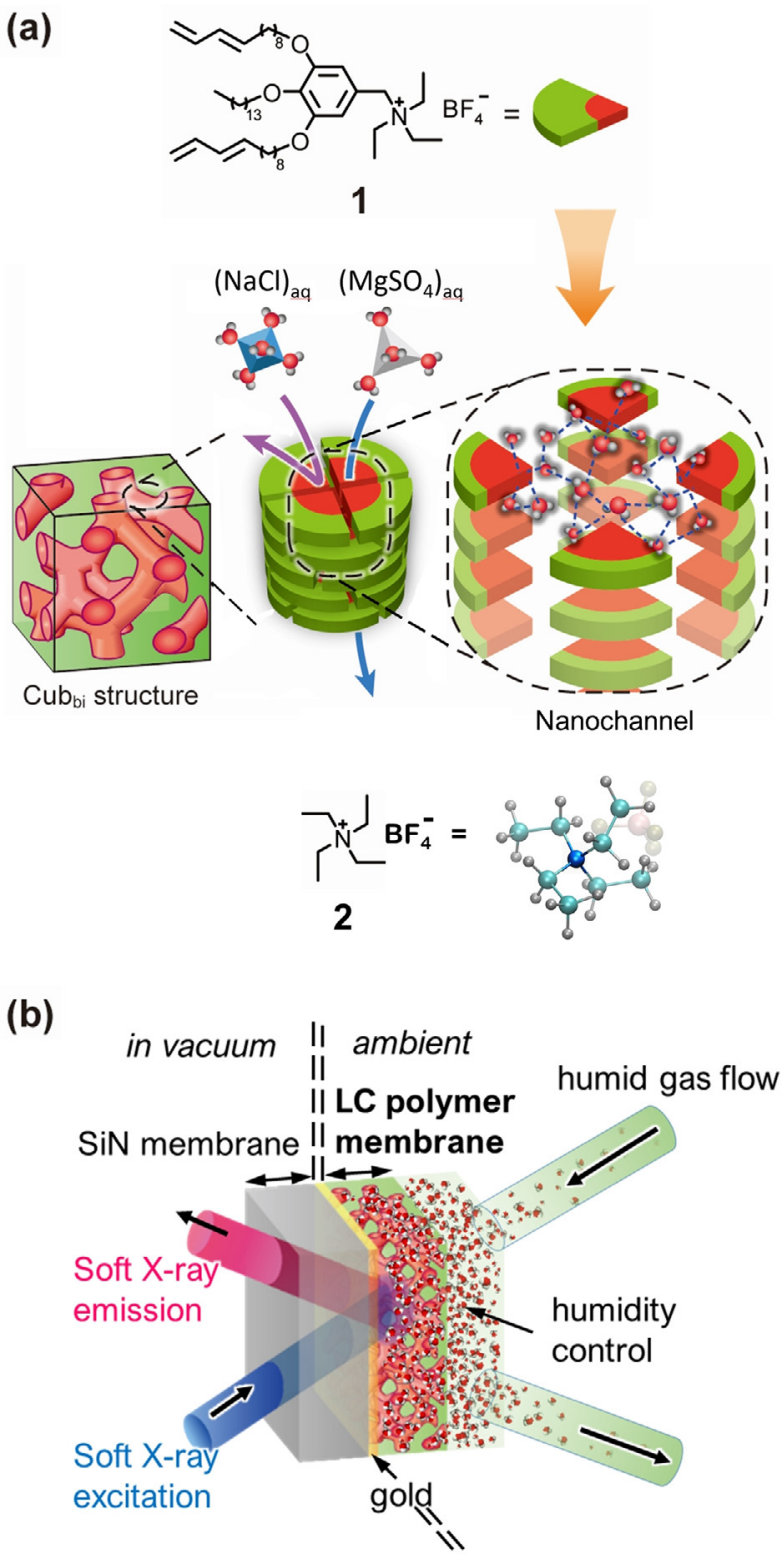
a) Molecular structure and illustration of an LC monomer benzyl triethylammonium tetrafluoroborate (**1**; top). The LC monomers were self‐assembled to form a bicontinuous cubic liquid‐crystal (Cub_bi_) structure (middle left). Ions are selectively transported (middle center) through subnanopores composed of a stack of the four LC monomers, where water forms specific hydrogen‐bonded structures (middle right). Tetraethylammonium tetrafluoroborate [N(C_2_H_5_)_4_BF_4_] (**2**) as a reference has the same moiety as compound **1** (bottom). b) Soft X‐ray emission detection from water in the LC membrane.

XES is a unique tool for probing the element‐specific valence electronic structure of materials.^[15]^ Figure 2 a displays how the valence electronic structure of water is obtained after oxygen 1s core excitation. Decay of the O 1s core hole by selective transition from the p‐symmetric 1b_2_, 3a_1_, and 1b_1_ valence states accompanies the X‐ray emission. Because the XES process obeys the strict symmetry selection rule, O 1s XES is very sensitive to changes in the local hydrogen‐bonded configuration of water.^[16]^ This is demonstrated in Figure 2 b by comparison of XES spectra for an isolated water molecule with a hydrogen‐bonded water molecule. The most striking change upon condensation from the gas phase to the liquid phase is the splitting of the single 1b_1_ peak in an H_2_O molecule to double 1b_1_′ and 1b_1_′′ peaks in liquid H_2_O. The 1b_1_′ peak position is smoothly connected to the corresponding peak in H_2_O ice[^17^, ^18^] and can be associated with tetrahedrally coordinated H_2_O molecules (effectively four hydrogen bonds per molecule). The 1b_1_′′ peak between the 1b_1_′ and the gas‐phase 1b_1_ peaks can be associated with highly distorted hydrogen‐bonded H_2_O molecules.[^18^, ^19^, ^20^] For aqueous salt solutions, the XES profile of water is further modulated due to a change in the hydrogen‐bonded structures of water molecules hydrating the solutes.[^21^, ^22^] This is demonstrated at the bottom of Figure 2 b and is discussed below in detail.

**Figure 2:**
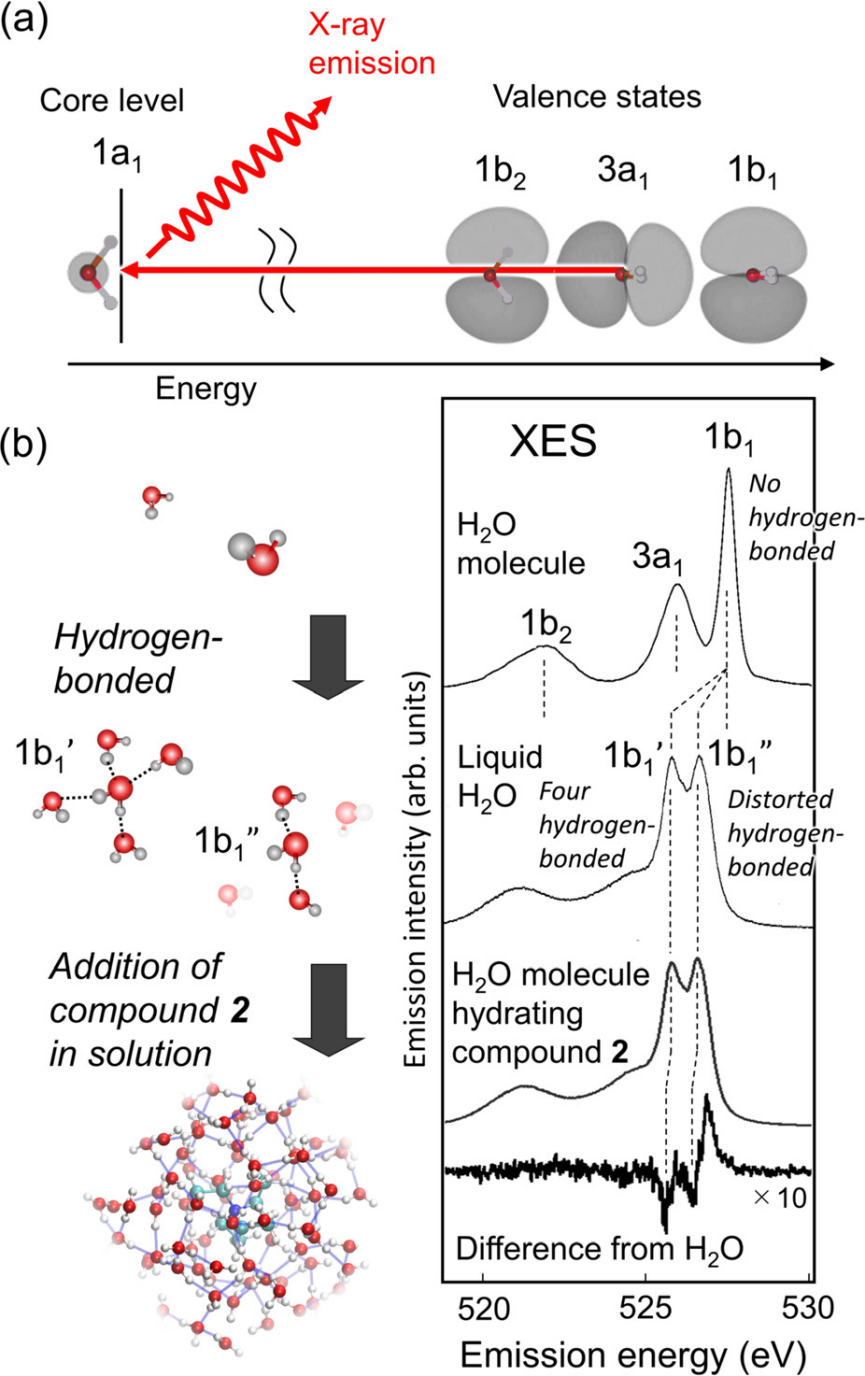
a) Valence electronic states of an H_2_O molecule probed by X‐ray emission spectroscopy. b) Three distinct 1b_1_, 3a_1_, and 1b_2_ orbitals of an H_2_O molecule (top) are significantly modified by the effect of hydrogen bonding in liquid H_2_O (middle). Interaction of an H_2_O molecule with 1 mol L^−1^ compound **2** [N(C_2_H_5_)_4_BF_4_] solution further modifies the energy of each orbital from liquid H_2_O, as demonstrated in the difference spectrum (bottom).

An LC polymer film was prepared from compound **1** by spin‐coating and photopolymerization (see Experimental details in the Supporting Information). Figure 1 a is a schematic drawing of an LC membrane with the Cub_bi_ phase. Water runs only through nanochannels with a pore size of ca. 0.6 nm,^[7a]^ which are formed by in situ polymerization of self‐assembly of **1**. We employed compound **2** bearing the triethylammonium moiety (also shown in Figure 1 a) as a model compound to obtain insights into the ionic interactions between the cationic moiety and the inorganic anions (SO_4_^2−^, Cl^−^) because this cationic moiety plays a key role in the nanochannel of **1**.

XES spectra of pure liquid H_2_O absorbed in the LC membrane and hydrating water of permeating solutes, MgSO_4_ and NaCl were collected at the SPring‐8 BL07LSU HORNET station using a high‐resolution XES spectrometer.^[23]^ A custom‐made ambient pressure cell was used to expose the LC membrane to humidity‐controlled moisture and nitrogen as a carrier gas. By carefully increasing the humidity, it was possible to control the accumulation of H_2_O molecules in the LC membrane (Figure 1 b).

XES measurements of the hydrating water in the MgSO_4_ and NaCl solutions were performed by simply circulating concentration‐controlled (1 mol L^−1^ and 3 mol L^−1^ for NaCl and 1 mol L^−1^ and 2.5 mol L^−1^ for MgSO_4_) solutions through the tube. One XES spectrum was obtained in a few hours by scanning approximately 100×100 μm^2^ area, giving time‐ and space‐averaged information for hydrogen‐bonded structure of water. Details of the experimental setup and operating condition are described elsewhere.^[24]^ All measurements were done at room temperature.

The O 1s XES spectra of the LC membrane (Figure 3, spectra **A**, **B**) are compared with bulk liquid H_2_O (Figure 3, spectrum **C**). First, we examined the contribution of ether O atoms in the LC membrane to the O 1s XES spectra by flowing dried N_2_ gas through the tube. With a relative humidity of 10 %, we obtained spectrum **A** in Figure 3. The relative area intensity of the XES spectra of the bulk liquid H_2_O to spectrum **A** is 6.1, as evaluated from Figure 3. This value is close to the expected relative XES intensity of 7.3 estimated from the experimental geometry (detailed estimates are discussed in the Supporting Information). Therefore, we conclude that spectrum **A** roughly represents the electronic structure of ether O atoms in the LC membrane. Next, to extract the electronic structure of water absorbed by the LC membrane, spectrum **A** was subtracted from the XES spectrum of the fully humidified (95 % relative humidity) LC membrane (spectrum **B′** in Figure S2), and the result is shown as spectrum **B** in Figure 3. The relative area intensity of spectrum **B** to spectrum **A** is 1.5, which is much larger than the expected intensity of 0.36 for water fully incorporated into the subnanopores (detailed estimates are discussed in the Supporting Information). The large signal intensity of spectrum **B** should be due to accumulation of bulk liquid H_2_O on the LC membrane after the subnanopores are filled with H_2_O molecules. With an equal integrated area intensity between 515 and 530 eV, spectrum **C** for the bulk liquid H_2_O was subtracted from spectrum **B** (Figure 4 a). The obtained profile directly reflects the modulation of the hydrogen‐bonded structures of hydrating water molecules from the bulk liquid H_2_O. The two significant valleys in the plot at the 1b_1_′ (525.7 eV) and 1b_1_′′ (526.5 eV) positions indicate the loss of water species specific to the bulk liquid H_2_O. The further decrease of the 1b_1_′ peak compared to the 1b_1_′′ peak implies the destruction of tetrahedrally (four‐fold) coordinated H_2_O molecules (Figure 2 b)[^15^, ^16^] in the LC membrane. Deconvolution of the 1b_1_ profile into Gaussian peaks (Figure 4 a) requires the presence of a broad positive peak Δ1b_1_^LC^ centered around the middle of the Δ1b_1_′ and Δ1b_1_′′ valleys. The Δ1b_1_^LC^ peak must represent the hydrogen‐bonded structures of water in the subnanopore of the LC membrane because the Δ1b_1_^LC^ peak compensates for the loss of the bulk liquid H_2_O components (Δ1b_1_′ and Δ1b_1_′′). The broad profile of the Δ1b_1_^LC^ peak implies the presence of several hydrogen‐bonded configurations of water in the subnanopores of the LC membrane.

**Figure 3:**
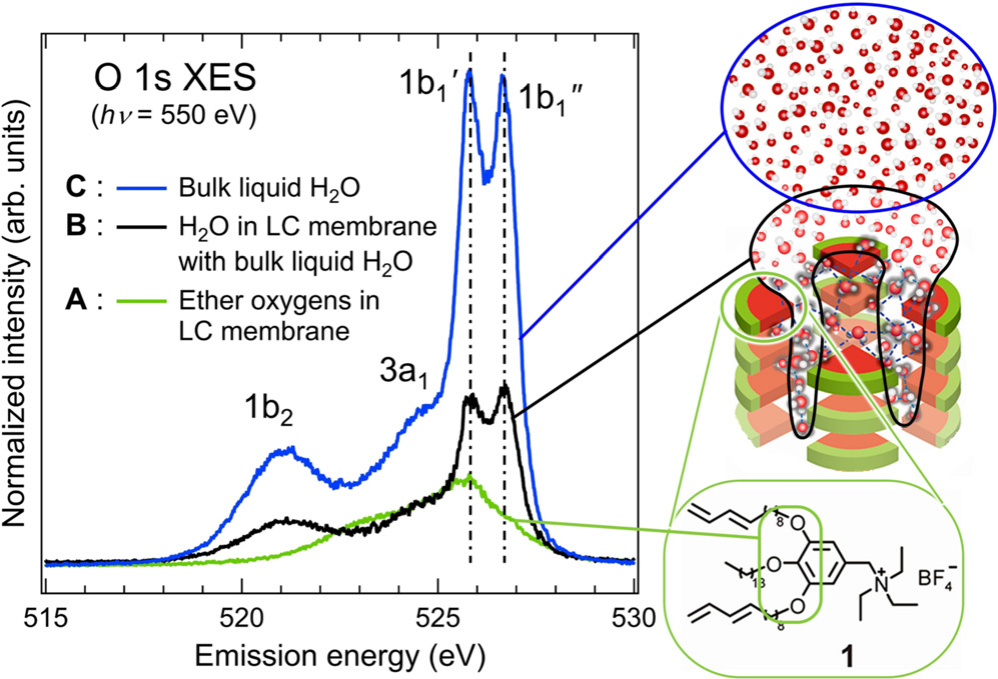
O 1s XES spectra of ether oxygen atoms in the LC membrane (**A**, green), H_2_O in the LC membrane with the bulk liquid H_2_O (**B**, black), and the bulk liquid H_2_O (**C**, blue). Relevant oxygen moieties in each spectrum are shown on the right.

**Figure 4:**
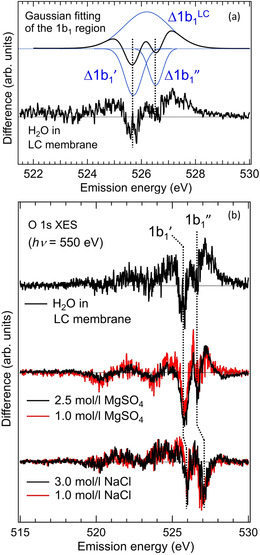
a) Modulation of the valence 1b_1_ structure of H_2_O in the LC membrane from bulk liquid H_2_O, and reproduction of the 1b_1_ profile by three Gaussian peaks (Δ1b_1_′, Δ1b_1_′′, and Δ1b_1_^LC^). b) Valence electronic structure of H_2_O in the LC membrane compared with 1.0 mol L^−1^ and 2.5 mol L^−1^ MgSO_4_, as well as 1.0 mol L^−1^ and 3.0 mol L^−1^ NaCl aqueous solutions.

To explore the origin of the selective ion permeation (73±5 % for MgSO_4_ and 30±6 % for NaCl), hydrogen‐bonded structures of H_2_O in the LC membrane were compared with those in the MgSO_4_ and NaCl hydrations (Figure 4 b). We measured XES for two different concentrations of each solute, 1 mol L^−1^ and 2.5 mol L^−1^ for MgSO_4_ and 1 mol L^−1^ and 3 mol/l for NaCl, to see if the concentration affects the hydrogen‐bonded structures of hydrating water. This is crucial for evaluating the stability of the selective ion permeation if the hydrogen‐bonded structures of water dominantly control the ion selectivity. The normalized difference XES spectra for the two solutes from bulk liquid H_2_O reflect the modulation of the hydrogen‐bonded structures of hydrating water (Figure 4 b).

In the MgSO_4_ solution, hydrating water has hydrogen‐bonded structures similar to those of water in the subnanopores of the LC membrane, which is significantly different from water in the NaCl solution. At least in the measured concentration range, it is found that the XES profile primarily depends on the ionic species and is not affected by the concentration of each salt. Below the critical concentration range around 3 mol L^−1^ for MgSO_4_ and 5 mol L^−1^ for NaCl, where the hydration spheres of cations and anions overlap and form contact ion pairs,[^25^, ^26^] hydrogen‐bonded structures do not change with concentration for each salt, which would also be responsible for the effective transportation of MgSO_4_ into the subnanopores of the LC membrane. Not only the XES profile that reflects the modulation of the hydrogen‐bonded structures, but we also evaluate the ratio of modulation compared with the net XES intensity for water molecules in the subnanopores of the LC membrane and hydrating water of the permeating solutes. By integrating the absolute value of the difference XES spectra in the displayed energy range from 515 eV to 530 eV (Figure 4 b), we found the ratio is 0.074 for water molecules in the subnanopore while 0.079 for water hydrating MgSO_4_ at 2.5 mol L^−1^. Intriguingly the extent of XES modulation is comparable between water molecules in the subnanopores of the LC membrane and hydrating water of MgSO_4_ near the critical concentration (3 mol L^−1^). This implies the hydrogen‐bonded structures of all the water molecules are modulated in the subnanopore with 0.6 nm diameter and at the critical concentration of MgSO_4_ solution.

From the above results, we conclude that the consistency of the hydrogen‐bonded structures of water in the subnanopores of the LC membrane and hydrating water contributes to the high permeability of the MgSO_4_ solution. The hydrogen‐bonded structures of water in the above two regions may affect the permeability of ions when the net gain in free energy as a sum of mixing enthalpy and mixing entropy is different. We expect the net gain is maximized when the two water regions have similar hydrogen‐bonded structures, although careful considerations of the free energy are necessary. The ion‐selective permeation should also be thermally controlled. At elevated temperatures, more distorted and less‐dense hydrogen‐bonded structures are expected for the hydrating water of MgSO_4_, while more fluctuation of the ionic groups will significantly perturb the hydrogen‐bonded structure of water in the subnanopore of the LC membrane. Consequently, the net loss of mixing entropy on temperature change must be studied. Theoretically, Tang and Kim investigated the temperature effects on the ion‐selective permeation of carbon nanotubes, and noted the possibility that the change in the hydration energy of ions for the temperature decrease of 150 K (from 450 K to 300 K) affected the ion‐selective permeation.^[27]^ However, the effect of thermal fluctuation may be small for carbon nanotubes. In the case of the LC membrane, it remains a challenge to understand the temperature effect on the ion‐selective permeation.

In the case of water molecules inside the subnanopores of the LC membrane, triethylammonium ionic groups covering the inside wall of the subnanopores neighbor a majority of the water molecules. Because this is dominated by the interaction between water and the alkyl chains connected to the charged nitrogen, we can compare the hydrogen‐bonded structures of water neighboring the triethylammonium cations by compound **2** through O 1s XES. Our difference XES spectrum of compound **2** against pure H_2_O water (Figure 2 b, bottom) shows a profile similar to that of water inside the subnanopores of the LC membrane (Figure 4 b). Slight differences to the right and left of the two 1b_1_ valleys can be explained by a blue shift of the Δ1b_1_^LC^ peak relative to the Δ1b_1_′ and Δ1b_1_′′ valleys. The difference of the Δ1b_1_^LC^ peak implies the presence of interference effects among hydrating water of the triethylammonium ionic groups in the quite limited space of the subnanopore.

In conclusion, we have studied the hydrogen‐bonded structures of water in the subnanopore of an LC membrane to explore the mechanism of its selective transport of ions, in particular the unexpected high permeability for large Mg^2+^ and SO_4_^2−^ ions. For the selective permeability of ions, we propose a possible contribution of structural consistency between the hydrogen‐bonded structures of water in the subnanopores and hydration layer surrounding the ions, in addition to the competition between the pore size and the ionic radius, the energy barrier for dehydration, and the electrostatic interaction between the charges in the subnanopore. The ion selectivity should appear as a result of the complicated intertwining of all the above properties, which are modulated by selecting the ionic groups that protrude into the subnanopores of the LC membrane. Water can be regarded as a part of the LC membrane contributing to its ion selectivity, which is similar to the perspective for the role of hydrating water that controls many functions of biological systems.

## Conflict of interest

The authors declare no conflict of interest.

## Supporting information

As a service to our authors and readers, this journal provides supporting information supplied by the authors. Such materials are peer reviewed and may be re‐organized for online delivery, but are not copy‐edited or typeset. Technical support issues arising from supporting information (other than missing files) should be addressed to the authors.

Supplementary
