# Supplementary material for: Ion Selectivity of Water Molecules in Subnanoporous Liquid‐Crystalline Water‐Treatment Membranes: A Structural Study of Hydrogen Bonding
Source: Angew Chem Int Ed Engl. 2020 Oct 19;59(52):23461–5. doi: 10.1002/anie.202008148 (PMC7756590; doi:10.1002/anie.202008148)
Supplement: Supplementary file 1 — Supplementary [file ANIE-59-23461-s001.pdf]

Angewandte Chemie
Eine Zeitschrift der Gesellschaft Deutscher Chemiker

Supporting Information

Ion Selectivity of Water Molecules in Subnanoporous Liquid-Crystalline Water-Treatment Membranes: A Structural Study of Hydrogen Bonding

Ryusuke Watanabe, Takeshi Sakamoto, Kosuke Yamazoe, Jun Miyawaki, Takashi Kato, and Yoshihisa Harada**

anie_202008148_sm_miscellaneous_information.pdf

Author Contributions

R.W. Data curation: Lead; Formal analysis: Equal; Writing—Original Draft: Supporting
T.S. Investigation: Equal; Methodology: Equal; Writing—Original Draft: Supporting
K.Y. Data curation: Supporting; Formal analysis: Supporting; Investigation: Supporting; Methodology: Equal
J.M. Data curation: Supporting; Formal analysis: Supporting; Methodology: Equal; Software: Supporting
T.K. Conceptualization: Lead; Funding acquisition: Lead; Methodology: Lead; Project administration: Lead; Resources: Lead; Supervision: Lead; Validation: Equal; Visualization: Equal; Writing—Original Draft: Equal; Writing—Review & Editing: Lead
Y.H. Conceptualization: Equal; Data curation: Equal; Formal analysis: Lead; Funding acquisition: Equal; Investigation: Equal; Methodology: Lead; Project administration: Lead; Resources: Equal; Supervision: Equal; Validation: Equal; Visualization: Equal; Writing—Original Draft: Lead; Writing—Review & Editing: Equal.

Experimental details

A solution of the organic precursor was prepared by mixing compound **1** and a photo-initiator, 2,2-dimethoxy-2-phenylacetophenone, in dichloromethane at concentrations of 2.0 wt% and 0.02 wt%, respectively. The solution was spin-coated at 700 rpm for 40 sec onto a 3 nm Au-coated silicon carbide (SiC) membrane 150 nm thick (NTT-AT Corp., Japan) and supported by a Si substrate 0.625 mm thick. Subsequently, the substrate was heated at 80 °C and then cooled to 10 °C. UV light was irradiated onto the substrate using a high-pressure mercury lamp (12–13 mW cm^{-2}) at 10 °C for 10 min under N_2 flow.

To allow incident and emitted soft X-rays to pass through and probe the sample, the Si substrate has an opening (0.3 mm × 3 mm in size) where only the free-standing SiC membrane exists. The SiC membrane was also used to separate the wet sample from the high vacuum of 10^{-6} Pa to transmit soft X-rays throughout the beamline. The membrane was exposed to humidity-controlled moisture supplied through a 2 mm Teflon-based inert tube connected to the steam generation equipment (HUM-1, Rigaku Corp., Tokyo, Japan) filled with ultrapure water (Direct-Q, Millipore Inc., Billerica, MA).

Simple estimate of relative O 1s XES intensities for absorbed water in the subnanopore of the LC membrane and for the bulk liquid H_2O to ether oxygen in the LC membrane

To estimate the ratio of each oxygen moiety, we simplified the LC membrane system by considering the spatial dimension described by Sakamoto *et al.*[1] (Figure S1): subnanopores having a radius of ca. 0.3 nm are formed by four LC monomers squarely facing each other. The total length of the LC channels within the film having a size of 1.0 μm^3 is calculated to be 1.3×10^5 μm. Then the number of oxygen atoms in water incorporated in the subnanopore of the 1.0 μm^3 LC membrane is calculated as

$$\underbrace{(1.3 \times 10^5 \times 10^{-6})}_{\text{(LC channel length)}} \times \underbrace{[\pi \times (0.3 \times 10^{-9})^2]}_{\text{(Channel sectional area)}} \times \underbrace{[(6.023 \times 10^{23})/18 \times 10^6]}_{\text{(Number of } H_2O \text{ molecules in } m^3)} \approx 1.23 \times 10^9 \text{ atoms/1.0 } \mu m^3.$$

By assuming that each LC monomer is stacked at 0.45 nm intervals and one LC monomer contains three oxygen atoms, we can calculate the number of constituent oxygen atoms in the 1.0 μm^3 LC membrane as follows:

$$\underbrace{(1.3 \times 10^5 \times 10^{-6}) / (0.45 \times 10^{-9})}_{\text{(Number of stacks in the 1.0-}\mu m^3\text{ LC membrane)}} \times \underbrace{3 \times 4}_{\text{(Number of oxygen atoms in each stack)}} \approx 3.47 \times 10^9 \text{ atoms/1.0 } \mu m^3.$$

Accordingly, the relative number of oxygen atoms in water incorporated into the subnanopores are compared to the constituent oxygen atoms in the LC polymer with the following calculation:

$(1.23 \times 10^9) / (3.47 \times 10^9) \approx 0.36.$

The number of oxygen atoms in the 1.0 μm^3 bulk liquid H_2O is calculated as follows:

$(10^{-6})^3 \times [(6.023 \times 10^{23})/18 \times 10^6] \approx 3.35 \times 10^{10}$ atoms/1.0 μm^3.

Accordingly, the relative number of oxygen atoms in bulk liquid H_2O compared to the constituent oxygen atoms in the LC polymer is calculated as follows:

$(3.35 \times 10^{10}) / (3.47 \times 10^9) \approx 9.65.$

In the O 1s XES experiment for bulk liquid H_2O, we used a silicon nitride 150 nm thick as a window material, while for the LC membrane, we used the SiC 150 nm thick. After accounting for transmission, the actual signal intensity ratio is modulated by a factor of 1.33.

Then, we expect a relative intensity of 9.65/1.33 ≈ 7.3.

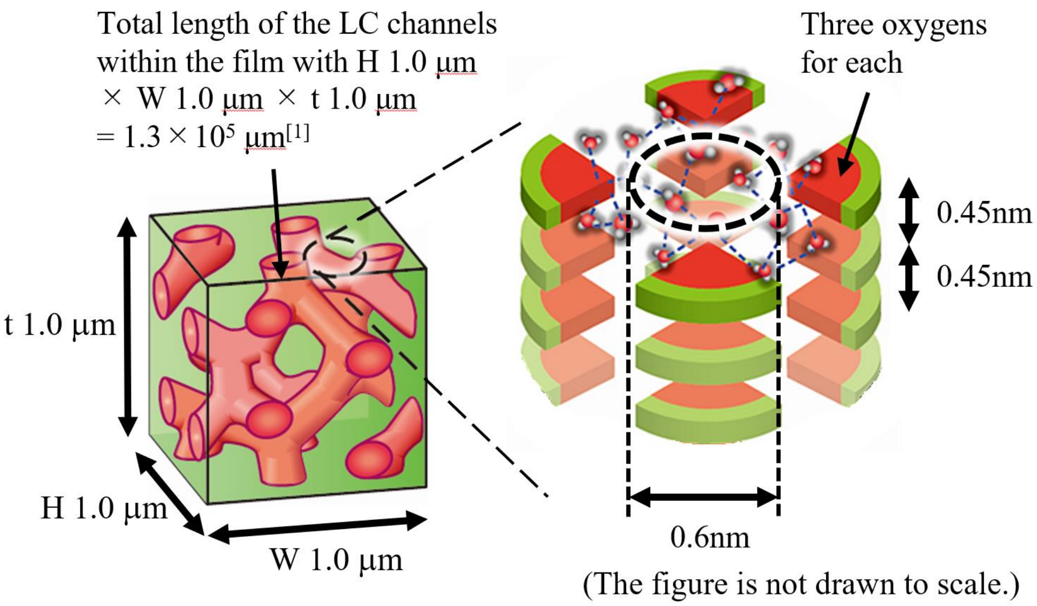

Figure S1. Geometrical configuration of the subnanopores in the LC membrane

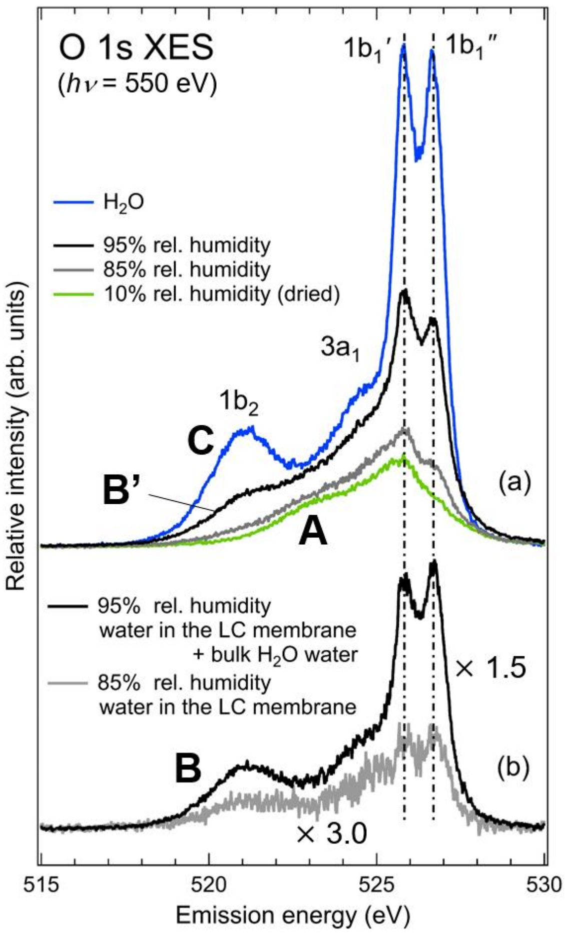

Figure S2. (a) O 1s XES spectra of the LC membrane under different relative humidity conditions – 10% (**A**, green), 85% (gray), and 95% (**B'**, black) – compared with bulk liquid H_2O (**C**, blue). (b) XES spectra of water in the LC membrane at 85% (gray) and 95% (**B**, black) relative humidity after subtraction of the dried spectrum representing water in the LC membrane (85%) and spectra overlapping with bulk liquid H_2O (95%).

The relative area intensity of the experimental XES spectra compared with that of the dried sample was 0.33 for the sample at 85% relative humidity. Thus, the LC membrane subnanopores are almost filled with water at 85% relative humidity. The effective filling of water in the subnanopores without applying pressure may be due to osmotic pressure and electrostatic interaction because the LC membrane has a charged ammonium ion in the side chain inside the wall of the subnanopores. After increasing the relative humidity above 85%, additional water is not incorporated into the subnanopores but is rather deposited on the LC membrane to form bulk liquid H_2O (as shown in the schematic image in Figure 3).

In Figure 3, we used the O 1s XES spectrum of the LC membrane in fully humidified condition (95% relative humidity) instead of the 85% condition that has a poor S/N ratio (Figure S2b). The use of the 95% spectrum is reasonable because the difference between the 85% and 95% relative humidity conditions is only the contribution from bulk liquid H_2O deposited on the LC membrane in the 95% humidity condition.

[1] T. Sakamoto, T. Ogawa, H. Nada, K. Nakatsuji, M. Mitani, B. Soberats, K. Kawata, M. Yoshio, H. Tomioka, T. Sasaki, M. Kimura, M. Henmi, T. Kato, *Adv. Sci.* **2018**, *5*, 1700405.
